# Fipronil Triggers Immunotoxicity Through Reactive Oxygen Species-Driven Mitochondrial Apoptosis in Thymocytes

**DOI:** 10.3390/toxics13030204

**Published:** 2025-03-12

**Authors:** Jui-Fang Kuo, Yai-Ping Hsiao, Yao-De Wang, Hsin-Pei Weng, Chia-Chi Wang

**Affiliations:** 1 School of Veterinary Medicine, National Taiwan University, Taipei 106, Taiwan; d06629001@ntu.edu.tw (J.-F.K.); poweredseven21@gmail.com (Y.-D.W.);; 2 Department of Biochemistry and Molecular Medicine, UC Davis Comprehensive Cancer Center, University of California Davis, Sacramento, CA 95817, USA; yshiau@ucdavis.edu

**Keywords:** fipronil, immunotoxicity, apoptosis, BCL-2 family, reactive oxygen species, mitochondrial membrane potential, glutathione, lipid peroxidation

## Abstract

Fipronil (FPN), a widely used pesticide, is associated with significant immunotoxic effects, particularly impacting thymocyte survival and immune homeostasis. This study explores the mechanistic pathways underlying FPN-induced apoptosis and oxidative stress. Short-term FPN exposure (1–10 mg/kg) notably suppressed the expression of both anti-apoptotic (*Bcl-2*, *Bcl-6*, *Mcl-1*) and pro-apoptotic (*Bnip3*, *Bim*) genes in thymic tissues in vivo. Additionally, in isolated primary thymocytes, FPN directly decreased the expression of *Bcl-2*, *Bcl-6*, *Mcl-1*, and *Bnip3* expression, coupled with a significant increase in pro-apoptotic *Bim* expression in a dose-dependent manner. FPN treatment directly led to elevated reactive oxygen species (ROS), lipid peroxidation, mitochondrial membrane depolarization, reduced cellular metabolic activity, and depleted intracellular calcium and glutathione (GSH) levels, indicating mitochondrial dysfunction and oxidative stress. Annexin V/PI staining confirmed that FPN induced late-stage apoptosis and necrosis in primary thymocytes. These findings elucidate the immunotoxic effects of FPN on thymocytes, highlighting its detrimental impact on immune system integrity, thymic development, and T cell maturation through oxidative damage and mitochondrial-mediated apoptosis.

## 1. Introduction

Fipronil (FPN) is known for its high specificity towards insect γ-aminobutyric acid (GABA_A_) receptors. The widespread use of FPN has consequently raised significant concerns about its harmful effects on non-target organisms. The contamination of water and soil by FPN and its metabolites poses an important and pressing public health and environmental concern [1,2,3]. FPN has been shown to cause significant mortality in aquatic organisms such as fish and amphibians, leading to disruptions in aquatic ecosystems [4]. Furthermore, evidence has shown that FPN exerts neurotoxic, hepatotoxic, and reproductive toxic effects on vertebrates [4,5,6,7,8,9,10,11,12]. Together, these findings underscore urgent public health concerns stemming from its unanticipated toxic effects.

The thymus serves as the primary lymphoid organ, integral to the maturation of T-cells and the establishment of immunological tolerance. It plays a pivotal role in the positive selection, ensuring T cells that recognize self-MHC molecules are retained, and in the negative selection, eliminating autoreactive T cells to prevent autoimmunity. Disruption of thymic homeostasis can significantly impair systemic immune function, increasing susceptibility to immunodeficiencies and autoimmune disorders. Our previous studies demonstrated that FPN induces immunotoxic effects in mature and immature T cells. FPN disrupts antigen-specific immune responses through the modulation of GABAergic signaling [13]. FPN has been shown to induce thymic atrophy and thymocyte depletion by modulating the IL-7 signaling pathway, a critical regulator of thymic development, and by disrupting the expression of transcription factors essential for T-cell survival and lineage commitment [14]. These findings strongly suggest that FPN exerts potent immunotoxic effects, potentially leading to immunosuppression. This highlights the critical need for further investigation into the precise mechanisms by which FPN impacts thymocyte homeostasis.

Apoptosis, or programmed cell death, is a key mechanism in sustaining cellular homeostasis, tissue integrity, and immune regulation [15]. The thymus removes dysfunctional or autoreactive thymocytes, thereby preserving immune tolerance and systemic integrity [16,17]. This mechanism depends on the regulation of the mitochondrial apoptotic pathway by the BCL-2 protein family. Anti-apoptotic BCL-2 family members, including BCL-2 and MCL-1, maintain mitochondrial integrity by inhibiting the pro-apoptotic proteins BAX and BAK. This inhibition prevents cytochrome *c* release and the subsequent activation of caspases. Conversely, pro-apoptotic proteins like Bim and bNIP3 facilitate mitochondrial membrane permeabilization in response to various cellular stressors [18]. Dysregulation of these pathways during thymic development can severely compromise immune homeostasis, potentially leading to immunodeficiencies or autoimmune conditions.

Prior research has demonstrated that FPN causes oxidative stress and a variety of off-target toxicities in animals, mostly due to an imbalance between reactive oxygen species (ROS) and reactive nitrogen species (RNS), leading to oxidative damage [6,8,19,20,21,22]. This stress compromises the antioxidant defense system, generating hydroxyl radicals that cause damage to cellular macromolecules [6,23]. Mice were orally administered FPN at 10 mg/kg (approximately 10% of the LD_50_) for 28 days. The liver, brain, and kidney tissues showed an apparent decline in the expression of antioxidant genes and a notable reduction in the activity of antioxidant enzymes including glutathione S-transferase (GST), superoxide dismutase (SOD), catalase (CAT), and glutathione (GSH) as a result of this exposure. FPN-induced lipid peroxidation was mitigated by supplementation with vitamin E or vitamin C [24,25]. These findings suggest that prolonged exposure to FPN induces oxidative stress, with lipid peroxidation likely serving as a key mechanism behind the oxidative damage observed. Meanwhile, ROS is crucial for T cell activation, expansion, and effector function interface [26,27,28]. Since thymocyte development relies heavily on ROS homeostasis [29], we focused on determining whether FPN exposure induces oxidative stress in thymocytes and exploring the underlying mechanisms of cell death.

Despite growing evidence of FPN’s broad-spectrum toxicity, the specific effects of FPN on thymocyte apoptosis and oxidative stress remain unclear. This study first investigates whether FPN alters apoptosis-regulating genes in vivo. Considering the continuous renewal and elimination of thymocytes within the thymus in vivo, it is challenging to directly observe the effects of toxicants on thymocyte death. Subsequently, in vitro experiments are conducted to explore the direct toxic effects of FPN on thymocyte death in the presence of the antioxidant N-acetylcysteine (NAC). This study aims to elucidate the complex interplay between oxidative stress and apoptosis in thymocytes, offering critical insights that could inform strategies to mitigate the immunotoxic effects of FPN exposure.

## 2. Materials and Methods

### 2.1. Chemicals and Reagents

Fipronil (FPN, 97%) was obtained from Tokyo Chemical Industry Co., Ltd. (Tokyo, Japan). RPMI 1640 medium (Cat. No. SH30027.02) was sourced from HyClone (Logan, UT, USA). Fetal bovine serum (FBS, Cat. No. 10437-028) and other cell culture reagents were provided by GIBCO BRL (Gaithersburg, MD, USA) and GE Healthcare (Chicago, IL, USA). Reagents for ELISA analysis were supplied by BD Biosciences (San Jose, CA, USA). All other reagents, unless otherwise specified, were obtained from Sigma (St. Louis, MO, USA).

### 2.2. Experimental Animals

Male BALB/c mice (3 weeks old, approximately 12–14 g body weight) were obtained from the BioLASCO Experimental Animal Center (BioLASCO, Taipei, Taiwan). Upon arrival, the mice were stratified by body weight to reduce initial weight variation and then randomly assigned to five groups. Each mouse was housed individually under controlled environmental conditions (temperature: 20–25 °C, humidity: 50–60%, 12-h light/dark cycle) with unrestricted access to food and water ad libitum. All animal experiments complied with the ethical guidelines of the Institutional Animal Care and Use Committee of National Taiwan University (IACUC Approval No: NTU108-EL-00026).

### 2.3. Protocol of Sub-Chronic Animal Experiment and Preparation of Thymocytes from Mice Thymus

**Figure 1:**
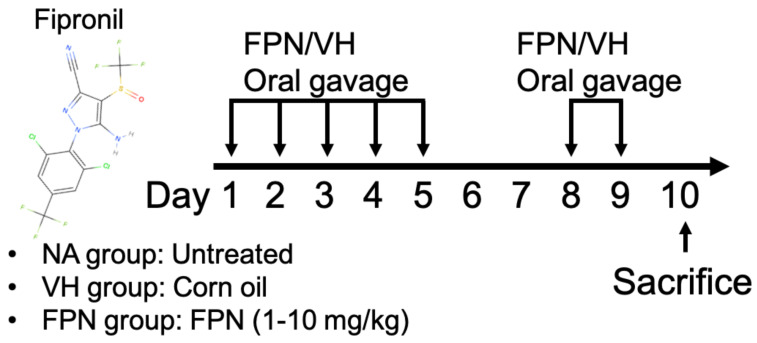
Protocol for fipronil (FPN) administration.

Four-week-old mice (five animals per group) were randomly assigned to one of five groups after one week of acclimatization (Figure 1). The highest dose of FPN, 10 mg/kg (equivalent to 1/10 of the oral LD_50_ in mice), was chosen based on previous studies to minimize the risk of acute toxicity and mortality while inducing sub-chronic toxic effects during the seven-dose treatment period [9,13]. To investigate dose-dependent effects, lower doses of 1 mg/kg (equivalent to 1/100 of the LD_50_) and 5 mg/kg (equivalent to 1/20 of the LD_50_) were conducted.

Following euthanasia, the thymus was harvested to assess oxidative stress. Each thymus was rapidly excised, mechanically dissociated, and processed into a single-cell suspension. The cells were cultured in RPMI-1640 medium supplemented with 5% heat-inactivated FBS in a 37 °C incubator with 5% CO_2_.

The mice were randomly divided into the naïve (NA), vehicle-treated (VH), and FPN-treated groups. The dosing regimen for FPN administration is described in Materials and Methods.

### 2.4. RNA Isolation and Quantitative Polymerase Chain Reaction (qPCR)

Total mRNA was extracted from thymus tissue and isolated thymocytes (stimulated with or without ConA for 24 h). The samples were homogenized using TRIzol reagent and purified with the GENEzol Pure Kit (Geneaid Biotech Ltd., New Taipei City, Taiwan) according to the manufacturer’s protocol. The extracted mRNA was then reverse-transcribed into cDNA, which was subsequently used for quantitative PCR (qPCR) as described previously. Gene expression levels were quantified using the ΔΔC_t_ method, with normalization to *Hprt* mRNA levels. The primers used for target gene analysis are listed in Table 1.

### 2.5. Evaluation of Mitochondrial Function

The mitochondrial metabolic activity of primary thymocytes following FPN treatment was analyzed using an MTT (3-[4,5-dimethylthiazol-2-yl]-2,5-diphenyltetrazolium bromide) assay. Thymocytes were seeded into a 96-well plate for 24 h. To assess cellular metabolism, the treated thymocytes were incubated with an MTT stock solution (5 mg/mL), enabling the conversion of MTT into formazan crystals, which served as an indicator of mitochondrial function. The purple formazan crystals were dissolved in dimethyl sulfoxide (DMSO), and the absorbance was recorded to determine cell viability [16]. After incubation, the absorbance was measured at 570 nm using an ELISA microplate reader (SpectraMax^®^ M5 Microplate Reader, Molecular Devices LLC, San Jose, CA, USA), with 630 nm used as a background reference to ensure accurate quantification.

### 2.6. Evaluation of Cytokines by Enzyme-Linked Immunosorbent Assay (ELISA)

Thymocytes were isolated and cultured at a concentration of 5 × 10^6^ cells/mL in 48-well plates, with 0.3 mL of cell suspension per well. Cultures were performed in quadruplicate. The cells were stimulated with PMA/Iono for 24 h. Following incubation, cell culture supernatants were collected, and IL-2 levels were quantified using a commercially available ELISA kit (BD Biosciences, San Jose, CA, USA). Optical density was measured at 450 nm using a SpectraMax^®^ M5 Microplate Reader (Molecular Devices LLC, San Jose, CA, USA).

### 2.7. Assessment of Apoptotic/Necrotic Indicators

Apoptosis in primary thymocytes following FPN treatment was evaluated using the Annexin V-FITC Apoptosis Detection Kit (Dojindo, Kumamoto, Japan) following the manufacturer’s protocol. A total of 1 × 106 primary thymocytes were harvested, resuspended in 100 µL of the staining solution, and incubated for 15 min at room temperature in the dark. After staining, flow cytometry (BD FACSCalibur, San Jose, CA, USA) was performed to analyze apoptosis, using a 488 nm laser for excitation. FITC fluorescence was detected through a 515 nm bandpass filter, while PI fluorescence was recorded using a filter > 600 nm.

### 2.8. Measurement of Mitochondrial Depolarization

The mitochondrial membrane potential (Δψm) of primary thymocytes was evaluated using the JC-1 detection kit (Invitrogen, Carlsbad, CA, USA) according to the manufacturer’s protocol. The JC-1 stock solution (2.5 mg/mL) was prepared in DMSO and diluted to prepare the working solution. Cells were harvested and incubated in the dark with the JC-1 working solution for 30 min at 37 °C. Fluorescence signals (red aggregates and green monomers) were measured using a flow cytometer.

### 2.9. Measurement of Intracellular Calcium

The intracellular calcium of primary thymocytes was evaluated using the Fluo-4 AM detection kit (Invitrogen, Carlsbad, CA, USA) according to the manufacturer’s protocol. The Fluo-4 AM stock solution was prepared in DMSO and diluted to prepare the working solution (final concentration: 1 μM). Cells were harvested and incubated in the dark with the Fluo-4 working solution containing 0.02% of the non-ionic detergent Pluronic ^®^F-127 for 30 min at 37 °C. Following incubation, excess Fluo-4 dye was removed by washing the cells, and calcium levels were subsequently analyzed using a flow cytometer.

### 2.10. Detection of Glutathione (GSH) Activity

Primary thymocytes’ intracellular glutathione (GSH) levels were measured using the fluorescent probe 5-chloromethyl fluorescein diacetate (CMFDA; CellTracker Green, Invitrogen). A CMFDA stock solution (10 mM) was prepared in DMSO. After harvesting, the cells were centrifuged to remove the supernatant, and the pellet was resuspended in the CMFDA working solution. The cells were incubated in the dark at 37 °C for 30 min. Following incubation, excess CMFDA dye was removed by washing the cells, and GSH levels were subsequently analyzed using a flow cytometer.

### 2.11. Quantification of Intracellular ROS Levels

The levels of intracellular ROS were assessed using 2′,7′-dichlorodihydrofluorescein diacetate (H_2_-DCFDA; Invitrogen). The FPN-treated primary thymocytes were incubated with 5 μM H_2_-DCFDA for 30 min at 37 °C. Following incubation, excess dye was removed by washing with warm PBS, and the cells were analyzed using a flow cytometer.

### 2.12. Evaluation of Lipid Peroxidation (LPO)

Lipid peroxidation in FPN-treated thymocytes was quantified using the C11-Bodipy^581/591^ fluorescent probe (Cayman Chemical, Ann Arbor, MI, USA). This sensor specifically detects LPO by undergoing oxidation in the presence of intracellular lipid peroxides, emitting a distinct bright green fluorescence signal that indicates oxidative stress. The cells were incubated with the working solution (final concentration: 20 μM) at 37 °C for 30 min in the dark. Post-incubation, fluorescence changes reflecting LPO levels were analyzed by flow cytometry.

### 2.13. Statistical Analysis

Statistical analyses were performed using GraphPad Prism version 9 (GraphPad Software, Inc., La Jolla, CA, USA). The data are presented as mean ± standard error of the mean (SEM) for each treatment group in individual experiments. To determine the effects of FPN compared to the vehicle control (VH) group, the data were analyzed using one-way analysis of variance (ANOVA) followed by Dunnett’s multiple comparisons test. A *p*-value < 0.05 was considered statistically significant. All analyses were conducted in a blinded manner.

## 3. Results

### 3.1. Deregulation of BCL-2 Protein Family Gene Expression by FPN In Vivo, Ex Vivo, and In Vitro

#### 3.1.1. FPN Significantly Attenuated Bcl-2 Family mRNA Expression in the Thymus

**Figure 2:**
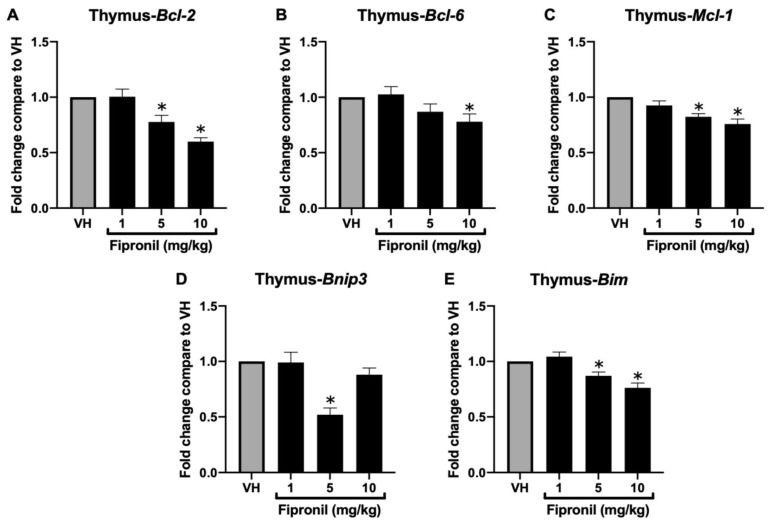
Fipronil significantly decreased mRNA expression of the *Bcl-2* family of thymus in vivo. The total mRNA harvested from different thymus treatment groups was extracted to detect the mRNA expression by qPCR. The expression level of *Hprt* was used as the control for semi-quantification. Results were expressed as the mean ± SEM pooled from four independent experiments with technological duplication in each group (N = 20/group). * *p* < 0.05 was significant compared to the VH group.

Our previous study demonstrated that sub-chronic exposure to FPN induced severe thymic atrophy in mice, accompanied by a substantial reduction in total thymocyte count. To further investigate the underlying mechanisms, we analyzed the expression of genes involved in the regulation of apoptosis and T-cell development. Total mRNA was extracted from thymus tissues to determine the expression levels of critical *Bcl-2* family genes involved in apoptosis regulation by qPCR, including the anti-apoptotic genes *Bcl-2*, *Bcl-6*, and *Mcl-1*, as well as the intrinsic pathway-associated pro-apoptotic gene *Bnip3* and *Bim* (Figure 2A–E). Following FPN administration, a significant reduction was observed in the mRNA expression of *Bcl-2*, *Mcl-1*, and *Bcl-6*, implicating a weakened anti-apoptotic defense. Interestingly, the expression of the pro-apoptotic gene *Bim* and *Bnip3* was significantly reduced as well.

#### 3.1.2. FPN Significantly Attenuated Bcl-2 Family mRNA Expression in ConA-Stimulated Thymocytes Ex Vivo

RNA analysis of whole thymic tissue extracts revealed significant downregulation of *Bcl-2* family target genes following FPN exposure. However, recognizing that whole thymic tissue comprises a heterogeneous population of cell types, we sought to minimize potential confounding effects arising from this cellular heterogeneity. To this end, mice were administered either VH or varying doses of FPN, and thymocytes were subsequently isolated and stimulated with concanavalin A (ConA) to assess the direct effects of FPN on these cells. Since in vivo FPN exposure may have already influenced thymocyte functions at the time of sampling, we aimed to investigate whether the thymocytes responding to ConA stimulation, which mimics T cell activation through T cell receptor (TCR) crosslinking, exhibit consistent alterations in apoptosis-related gene expression. By analyzing the expression of key apoptotic regulators in ConA-stimulated thymocytes, we aimed to determine if the transcriptional changes observed in vivo persist under ex vivo activation, potentially indicating dysregulation of survival pathways following FPN exposure.

**Figure 3:**
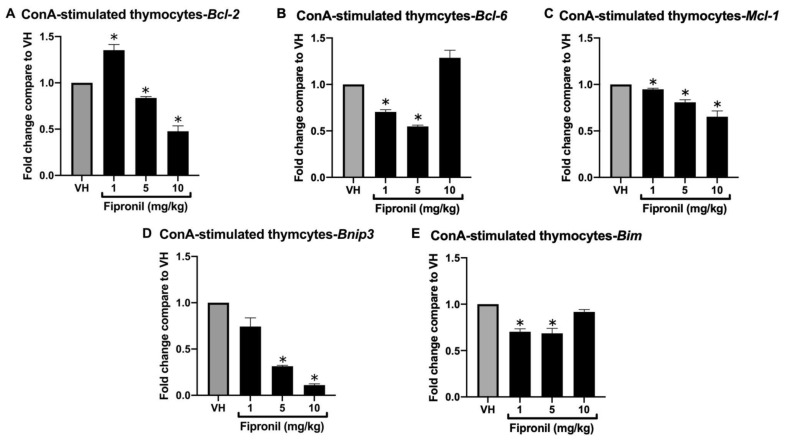
Fipronil significantly decreased mRNA expression of the *Bcl-2* family ex vivo. Total RNA was extracted from primary thymocytes following ConA stimulation to quantify mRNA expression by qPCR. The expression level of *Hprt* was used as the control for semi-quantification. Results were expressed as the mean ± SEM pooled from four independent experiments with technological duplication in each group (N = 20/group). * *p* < 0.05 was significant compared to the VH group.

Primary thymocytes were isolated and stimulated with ConA (final concentration, 5 μg/mL) for 24 h to further evaluate the impact of FPN on *Bcl-2* family gene expression in a more homogenous cell population. ConA, a mitogen that induces T-cell proliferation by cross-linking cell surface glycoprotein receptors, activates intracellular signaling pathways and transcription factors, thereby promoting cell cycle progression. Consistent with the results obtained from whole thymic tissue, high-dose FPN treatment significantly reduced the mRNA expression of *Bcl-2*, *Bcl-6*, *Mcl-1*, *Bnip3*, and *Bim* compared to the vehicle control (Figure 3A–E).

#### 3.1.3. Acute Exposure of FPN Significantly Attenuated Anti-Apoptotic mRNA Expression in the Primary Thymocytes In Vitro

**Figure 4:**
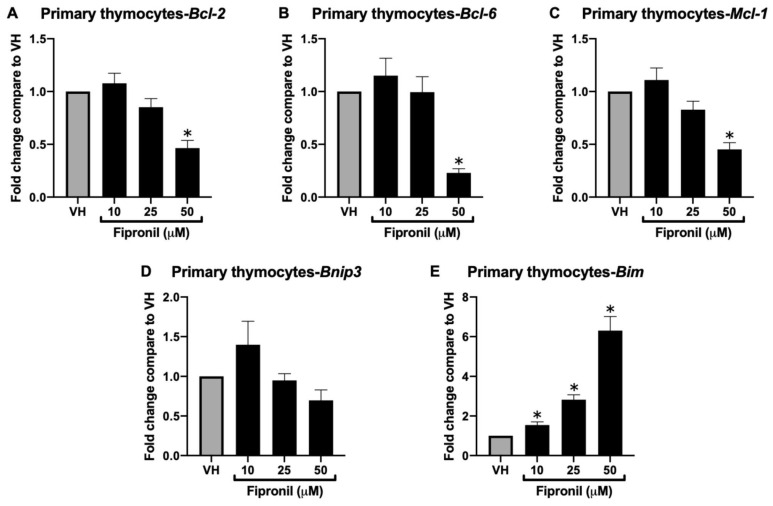
Fipronil significantly decreased mRNA expression of the *Bcl-2* family in vitro. The total mRNA harvested from different treatment groups was extracted to detect the mRNA expression by qPCR. The expression level of *Hprt* was used as the control for semi-quantification. Results were expressed as the mean ± SEM pooled from four independent experiments with technological duplication in each group. * *p* < 0.05 was significant compared to the VH group.

Our previous sub-chronic in vivo studies demonstrated that FPN exposure induces thymic atrophy and disrupts the expression of genes and proteins essential for T cell development and maturation. To further explore the underlying cellular and molecular mechanisms, we used an in vitro acute exposure model by treating isolated primary thymocytes with FPN. The anti-apoptotic capacity of primary thymocytes was also evaluated by assessing the mRNA expression. The analysis revealed a significant reduction in the expression of *Bcl-2*, *Bcl-6*, and *Mcl-1* genes following FPN treatment compared to the vehicle control, the same as the qPCR results of the in vivo model. In contrast, the expression of the pro-apoptotic gene *Bim* was significantly upregulated in a dose-dependent manner (Figure 4A–E). These findings highlight that FPN exposure disrupts multiple apoptotic regulatory mechanisms, not only diminishing the anti-apoptotic capacity of thymocytes but also selectively influencing pathways associated with mitochondrial integrity and stress-induced apoptosis.

### 3.2. Cytotoxic and Apoptotic Effects by FPN in an Acute In Vitro Model

#### 3.2.1. Cytotoxic and Immunosuppressive Effects of FPN on Primary Thymocytes

**Figure 5:**
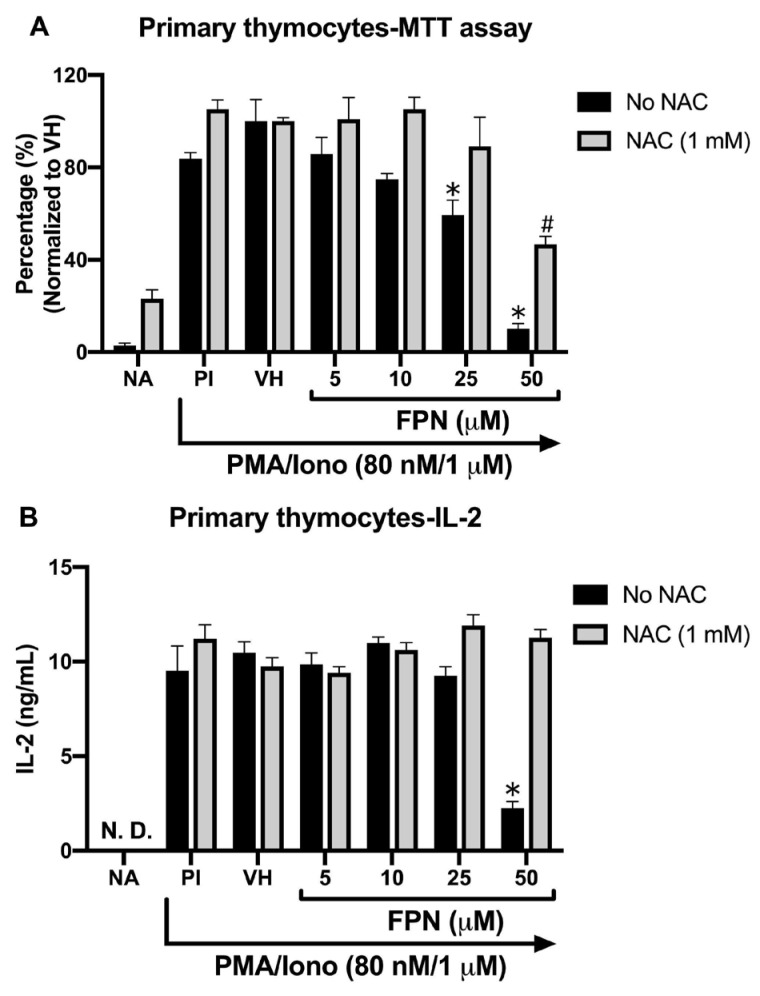
Fipronil exhibited a reduction of primary thymocyte viability and IL-2 production stimulated by PMA/Iono. The primary thymocytes (5 × 10^6^ cells/mL) were treated with 0.05% DMSO (VH) or FPN in different concentrations and stimulated with PMA/Iono (PMA/Iono: 80 nM/1 μM) for 24 h. (**A**) The viability of thymocytes was measured by MTT assay. (**B**) The level of IL-2 in the supernatants was measured by ELISA. Data were expressed as the mean ± SEM of quadruplicate cultures and representative of four independent experiments. * *p* < 0.05 was significant compared to the VH without the NAC group. # *p* < 0.05 was significant compared to the VH with the NAC group.

The cytotoxic effect of FPN on primary thymocytes was assessed using the MTT assay under PMA/Iono stimulation. Ionomycin facilitates Ca^2+^ release from the endoplasmic reticulum (ER), activating Ca^2+^-sensitive enzymes and synergizing with PMA to enhance protein kinase C (PKC) activation. This combined action effectively induces T cell activation, proliferation, and cytokine production, providing a robust model for studying T cell function and signaling pathways. After being treated with FPN at concentrations of 5, 10, 25, and 50 μM, the thymocyte viability was significantly reduced in a dose-dependent manner (Figure 5A). Similarly, under PMA/Iono stimulation, high concentrations of FPN (50 μM) significantly decreased IL-2 secretion (Figure 5B). Supplementation with N-acetylcysteine (NAC, final concentration: 1 mM) effectively reversed the toxic effects induced by FPN.

#### 3.2.2. Effects of FPN Treatment on Apoptosis in the Primary Thymocytes In Vitro

**Figure 6:**
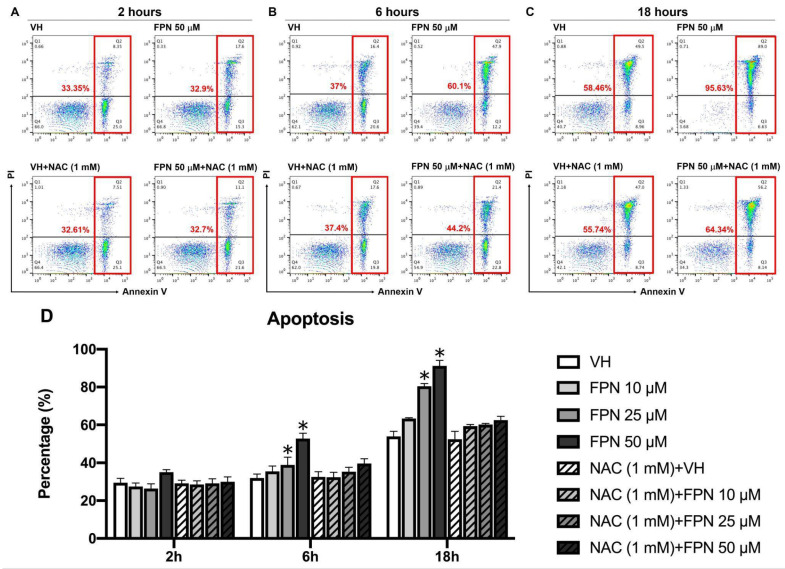
Fipronil inducted of thymocyte apoptosis in vitro. The apoptosis indicator was measured by Annexin V/PI staining. (**A**–**C**) The representative dot plot shows either the VH group and 50 μM FPN with or without NAC treatment at different time points. (**D**) Statistical data represent the sum of Annexin V^+^/PI^+^ and Annexin V^+^/PI^−^ populations. The primary thymocytes (5 × 10^6^ cells/mL) were treated with 0.05% DMSO (VH) or FPN in different concentrations for 2, 6, and 18 h. Data were expressed as the mean ± SEM of quadruplicate cultures and representative of four independent experiments. * *p* < 0.05 was significant compared to the VH group of each time point.

Apoptosis was evaluated using Annexin V/PI double staining. As shown in Figure 6A–C, cells in the Q4 quadrant (Annexin V^−^/PI^−^) represent viable thymocytes with minimal Annexin V and PI binding, indicating intact cell membranes. In contrast, cells in the Q2 and Q3 quadrants (Annexin V^+^/PI^+^ and Annexin V^+^/PI^−^) correspond to populations undergoing early and late stages of apoptosis, respectively. Our findings revealed a concentration- and time-dependent increase in thymocyte apoptosis following FPN exposure (Figure 6).

### 3.3. Mitochondrial Dysfunction and Oxidative Stress Dysregulation Induced by FPN Exposure in an Acute In Vitro Model

#### 3.3.1. Induction of Mitochondrial Depolarization on Primary Thymocytes by Fipronil

**Figure 7:**
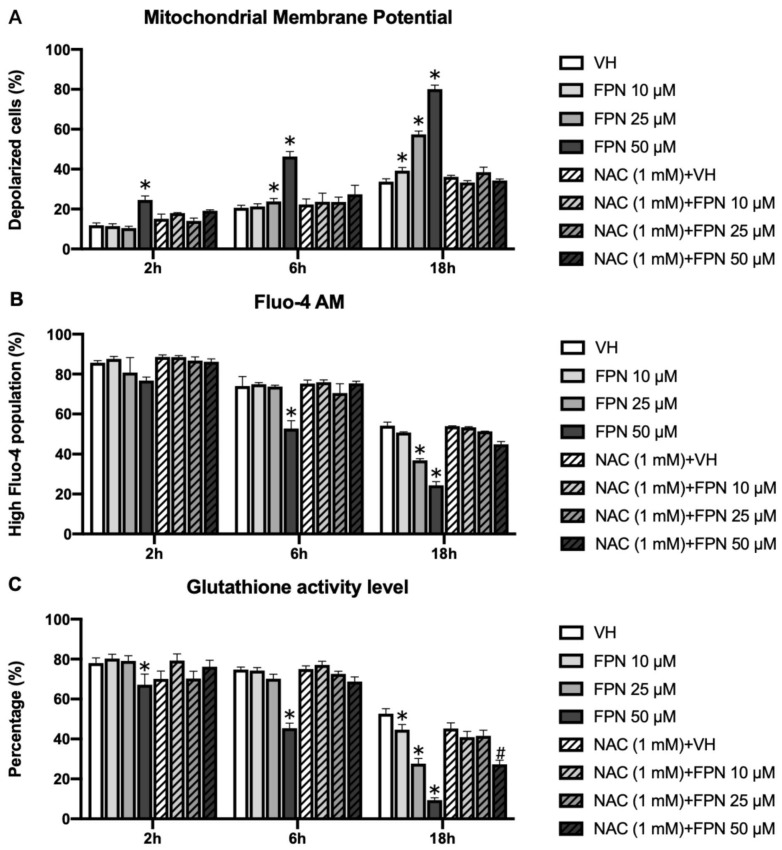
Effects of FPN on mitochondrial membrane potential, intracellular calcium levels, and GSH levels in primary thymocytes. Primary thymocytes (5 × 10^6^ cells/mL) were treated with 0.05% DMSO (VH) or FPN at different concentrations for 2, 6, and 18 h. (**A**) Mitochondrial membrane potential was assessed using JC-1 staining, (**B**) intracellular calcium levels were measured using Fluo-4 AM staining, and (**C**) intracellular GSH levels were determined using CMF-DA staining. Data are expressed as the mean ± SEM of quadruplicate cultures and are representative of four independent experiments. * *p* < 0.05 compared to the VH group of each time point. # *p* < 0.05 compared to the VH with the NAC group.

Mitochondrial depolarization was evaluated using JC-1 staining. In the primary thymocytes treated with 50 μM FPN, a significant increase in the proportion of depolarized cells was observed starting at 6 h. Additionally, starting from 18 h, a marked increase in mitochondrial depolarization was also evident in the cells treated with 10–50 μM FPN (Figure 7A).

#### 3.3.2. Depletion of Intracellular Calcium on Primary Thymocytes by Fipronil

To investigate potential disruptions in calcium homeostasis resulting from mitochondrial membrane potential loss, intracellular calcium levels were assessed using Fluo-4 AM staining. In the primary thymocytes treated with 50 μM FPN, a significant reduction in the level of calcium was observed starting at 6 h. Additionally, starting from 18 h, a marked decrease in calcium level was also evident in the cells treated with 10–50 μM FPN (Figure 7B).

#### 3.3.3. Reduction of Glutathione by Fipronil on Primary Thymocytes

The antioxidant glutathione (GSH) was assessed using CellTracker CMF-DA staining. The results indicated a significant decrease in GSH levels in the 50 μM FPN treatment group over a time course of 2 to 18 h, and the lower doses of FPN (10 and 25 μM) were decreased starting at 18 h (Figure 7C), which were consistent with the results of Annexin V/PI. This reduction in GSH activity indicated an increase in oxidative stress within the cells, suggesting that FPN exposure disrupted the redox balance. The supplementation of the antioxidant N-acetylcysteine (NAC, final concentration: 1 mM) effectively mitigated FPN-induced mitochondrial membrane potential changes, calcium, and GSH depletion.

#### 3.3.4. Accumulation of Intracellular ROS by Fipronil on Primary Thymocytes

**Figure 8:**
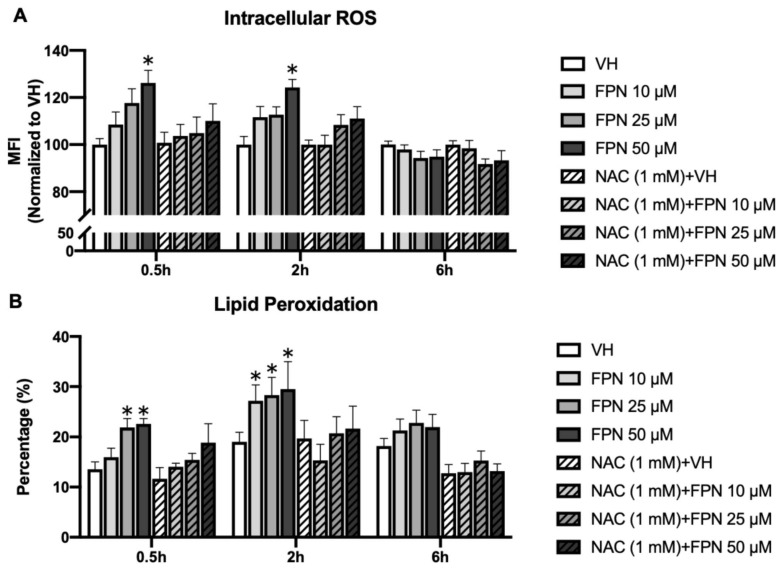
Induction of ROS and LPO levels by FPN. Primary thymocytes (5 × 106 cells/mL) were treated with 0.05% DMSO (VH) or FPN at different concentrations for 0.5, 2, and 6 h. ROS levels were measured using H₂-DCFDA staining, while LPO was assessed using C11-Bodipy581/591 staining. Data were expressed as the mean ± SEM of quadruplicate cultures and representative of four independent experiments. * *p* < 0.05 was significant compared to the VH group of each time point.

The levels of intracellular ROS were determined by the H_2_-DCFDA detection kit. The results revealed a marked H_2_O_2_ accumulation in the 50 μM FPN treatment group at an early point of 0.5 to 2 h (Figure 8A). After six hours, the effects of increased oxidative damage by FPN are no longer apparent. The treatment of NAC could prevent the oxidative stress induced by FPN.

#### 3.3.5. Fipronil Exposure Elevates Lipid Peroxidation in Primary Thymocytes

To characterize the ROS induced by FPN, lipid peroxide (LPO) levels in FPN-treated primary thymocytes were quantified using the fluorescent probe C11-Bodipy^581/591^. This sensor specifically detects LPO by undergoing oxidation in the presence of intracellular lipid peroxides, emitting a distinct bright green fluorescence signal that indicates oxidative stress. As shown in Figure 8B, a high concentration of FPN treatment resulted in a corresponding increase in lipid peroxidation levels at an early time point (0.5 h).

## 4. Discussion

Our sub-chronic in vivo and acute in vitro models provide the first investigation into the mechanisms underlying the immunotoxic effects of FPN on the thymus and primary thymocytes. While prior research has demonstrated that FPN induces oxidative stress and disrupts antioxidant defense mechanisms in various organs, its direct impacts on immune cells, particularly thymocytes, remain largely unexplored. Unlike hepatocytes, neurons, or renal cells, thymocytes are uniquely susceptible to oxidative stress due to their high turnover rate and critical role in T cell selection. Dysregulation of thymocyte apoptosis can have profound consequences on immune function, potentially increasing susceptibility to infections, altering immune tolerance, and predisposing individuals to autoimmune disorders. Our study reveals that even acute FPN exposure triggers oxidative stress-related apoptosis in thymocytes, which may contribute to persistent immune dysfunction.

FPN’s toxic effects are not limited to a single organ system, impacting key regulators of systemic homeostasis such as the liver, thyroid, kidneys, and central nervous system. These multiorgan effects can have cascading consequences, potentially contributing to the development of immunotoxicity. Hepatic metabolism of FPN via cytochrome P450 enzymes leads to the formation of fipronil sulfone, a more persistent and biologically active metabolite. This bioactivation process can induce oxidative stress and disrupt detoxification pathways in the liver, ultimately influencing systemic immune responses [30,31]. FPN has also been shown to disrupt thyroid hormone homeostasis by modulating thyroid-stimulating hormone (TSH) and thyroid peroxidase activity [19]. Thyroid hormones play a crucial role in immune function, regulating T cell maturation and differentiation. Therefore, FPN-induced alterations in thyroid hormone levels could contribute to immune dysregulation. Furthermore, FPN acts as an antagonist of GABAA receptors, disrupting neuronal signaling and potentially leading to neurotoxicity [20,21]. Given the intricate interplay between the immune and nervous systems, mediated by cytokines and neurotransmitters, the neurotoxic effects of FPN could contribute to immune dysregulation.

Given FPN’s widespread environmental persistence and presence as a residue on food products, its immunotoxic potential warrants further investigation. While previous research on FPN toxicity has mainly focused on active inflammatory responses and antigen-specific immune dysregulation [13,32], our prior findings demonstrated that FPN exposure severely impaired thymic development, disrupted IL-7 signaling, and led to significant thymic atrophy with marked reductions in thymocyte populations. To elucidate the underlying cellular and molecular mechanisms, we utilized both sub-chronic (young BALB/c mice) and acute (primary thymocyte cultures) models. These complementary approaches allowed us to dissect the immunotoxic effects of FPN at both the organismal and cellular levels, highlighting its potential risk in early-life exposure or acute poisoning events.

In our sub-chronic in vivo study, we opted for 4-week-old mice, a developmental stage characterized by active thymic function and ongoing T cell development. This age is particularly relevant for investigating potential immunotoxic effects, as the thymus plays a crucial role in immune system maturation. Furthermore, age-related differences in CYP enzyme activity can significantly impact the metabolism and toxicity of xenobiotics. Juvenile mice generally exhibit lower basal CYP activity compared to adults, potentially leading to slower FPN metabolism and prolonged systemic exposure [31]. This is particularly relevant given the link between CYP isozyme activity, oxidative stress, and apoptosis. Increased CYP-mediated metabolism can generate excessive ROS, potentially disrupting redox homeostasis and triggering apoptotic pathways. Considering the role of oxidative stress in FPN-induced immunotoxicity, metabolic differences between juvenile and adult mice could influence their susceptibility to FPN-induced immune dysregulation. Regarding dosage, our in vivo model employed a maximum FPN dose of 10 mg/kg. This is considerably lower than the estimated doses in reported cases of acute human FPN exposure (approximately 183–307 mg/kg) [33]. Even without considering interspecies differences in sensitivity and metabolic activity, our chosen dose allows for the investigation of potential immunotoxic effects at levels relevant to environmental or occupational exposures. Other in vitro studies using such neurons and hepatocytes have reported toxic effects of FPN within a concentration range of 30–200 μM [22]. The chosen concentrations of FPN in this in vitro study were within this range. While these concentrations may exceed those typically encountered in human exposure scenarios, they allow for the investigation of FPN’s toxic mechanisms in a controlled setting. Although our study may not directly reflect the concentrations that cause toxicity under typical exposure conditions, we have confirmed the involvement of apoptotic mechanisms in FPN-induced immunotoxicity both in vivo and in vitro.

Our findings demonstrated that administration of FPN (1–10 mg/kg) over seven doses to young mice significantly decreased the mRNA expression of the anti-apoptotic genes *Bcl-2*, *Bcl-6*, and *Mcl-1* (Figure 2 and Figure 3), indicating a disruption of anti-apoptotic mechanisms crucial for thymic homeostasis. Apoptosis plays a critical role in T cell biology, ensuring proper development, function, and immune homeostasis. During thymic development, thymocytes expressing nonfunctional or autoreactive T cell receptors (TCRs) are selectively eliminated via apoptosis, preventing the emergence of self-reactive cells that could induce autoimmunity. Furthermore, apoptosis regulates the contraction of expanded effector T cell populations following immune responses, thereby maintaining immune balance [34].

One major pathway leading to apoptosis is the intrinsic cell death pathway, controlled by Bcl-2 family members, which regulates mitochondrial membrane integrity. Both BCL-2 and MCL-1 are critical for T lymphocyte development and survival. BCL-2 production is tightly regulated during T-cell development to prevent abnormal apoptosis. Indeed, Bcl-2-deficient mice exhibit significant defects in T lymphocyte development, likely due to increased apoptosis [35,36]. In chimeric mice reconstituted with Bcl-2^−/−^ adult bone marrow hematopoietic stem cells, the development of donor-derived lymphocytes is almost absent [37]. Interleukin-7 (IL-7) provides a crucial survival signal for lymphocyte precursors, and Bcl-2 is believed to function as a key anti-apoptotic molecule downstream of IL-7 signaling [38,39]. Early deletion of Mcl-1 during T lymphocyte development results in a blockade of T-lineage cells at the DN stage, and its deficiency also leads to apoptosis in mature T lymphocytes. Furthermore, IL-7 signaling significantly upregulates Mcl-1 expression, indicating that Mcl-1 is a critical pro-survival molecule downstream of IL-7 signaling [40]. Hematopoietic stem cells (HSCs) also require Mcl-1 for their survival, and stem cell factor (SCF) signaling markedly enhances Mcl-1 expression in mouse HSCs [41]. Thus, Mcl-1 is an essential anti-apoptotic protein in lymphocytes and HSCs.

Interestingly, we also observed a reduction in pro-apoptotic genes, including *Bnip3* and *Bim*, which underscores a complex interplay in the apoptotic pathways. Bim is a key mediator of negative selection in the thymus, facilitating the elimination of autoreactive or dysfunctional thymocytes. The downregulation of Bim mRNA in vivo suggests a potential impairment in the negative selection process during T cell lineage commitment, which may disrupt the progression of thymocytes beyond the double-positive (DP) stage [42]. This finding is consistent with our previous study using the same sub-chronic exposure model, where FPN treatment resulted in a significant accumulation of DP thymocytes within the CD4/CD8 subpopulations [14]. Meanwhile, *Bnip3* plays a dual role in regulating mitochondrial dynamics and T-cell homeostasis [43]. Beyond its established function in hypoxia-induced apoptosis, *Bnip3* modulates mitochondrial membrane potential and turnover through mitophagy, ensuring the removal of damaged mitochondria and maintaining cellular health [44,45]. Its reduced expression may represent an adaptive response to FPN-induced oxidative stress, preserving mitochondrial function under adverse conditions. Therefore, simultaneous deregulation of pro-apoptotic and anti-apoptotic gene expression may impair thymocyte development, potentially leading to long-term immune deficiencies. These findings highlight the need for further investigation into the molecular mechanisms driving FPN-induced immunotoxicity and its broader implications for immune system health.

Contrary to the results of the reduction of *Bim* in the thymus of FPN-treated mice, FPN directly induced the *Bim* mRNA expression in a dose-dependent manner in vitro. Bim is a pro-apoptotic BH3-only protein that plays a central role in intrinsic apoptosis, particularly during thymocyte selection [46,47]. Its upregulation in vitro suggests that FPN directly induces apoptotic stress within thymocytes, potentially through oxidative or mitochondrial pathways. This outcome aligns with the observed oxidative damage in the lipid peroxidation assays and reduced GSH levels, which likely contribute to apoptotic signaling activation. The upregulation of Bim in vitro may result from the rapid cytotoxic effects of FPN, leading to mitochondrial-driven apoptosis and oxidative stress. Alternatively, this increase may reflect an attempt to eliminate damaged or stressed thymocytes, preventing the persistence of compromised cells. However, the differences between the in vitro and in vivo findings suggest additional factors influencing Bim expression within the thymic microenvironment. In the sub-chronic model, the suppression of Bim expression could be a response to limit excessive thymocyte loss, given the overall toxicity of FPN on the thymus. Another possibility is that surviving thymocytes in vivo may have already undergone selection pressure, favoring cells that downregulate Bim to resist apoptosis. These findings highlight the complexity of thymocyte responses under toxicant exposure and suggest that multiple factors contribute to the regulation of Bim expression in different experimental settings.

These findings underscore the dynamic interplay between intrinsic thymocyte responses and extrinsic regulatory signals in maintaining immune homeostasis under FPN exposure. While acute exposure rapidly activates apoptosis via oxidative stress pathways, prolonged exposure may induce adaptive changes that modify apoptotic sensitivity. Further investigations into thymic niche signaling and epigenetic regulation of pro-apoptotic genes will be crucial in understanding how FPN shapes thymocyte fate and immune system development over time.

As intrinsic apoptotic genes were altered during FPN exposure, we further evaluated the kinetic changes of mitochondrial damage and redox factors associated with FPN-induced thymocyte apoptosis. Under normal conditions, mitochondria convert approximately 1–2% of the total oxygen consumed into superoxide anions and other reactive oxygen species (ROS). However, exceeding ROS production may significantly induce intrinsic apoptosis during toxicant exposure or unhealthy cellular conditions. Consequently, mitochondria serve as the primary source of ROS generation within cells [48]. The detailed mechanisms of FPN-induced mitochondrial toxicity were investigated by assessing mitochondrial metabolic activity, mitochondrial membrane potential, and markers of oxidative damage, including ROS levels, lipid peroxidation, and intracellular GSH, in isolated primary thymocytes. The MTT assay demonstrated a significant reduction in the viability of primary thymocytes (Figure 5A), indicating that FPN treatment adversely affected mitochondrial metabolic activity. To further investigate the underlying mechanisms, we evaluated apoptosis and mitochondrial function using Annexin V/PI staining, JC-1 staining, intracellular calcium, and cellular glutathione levels, respectively. Our results revealed a dose- and time-dependent increase in the proportion of depolarized thymocytes at higher concentrations of FPN (Figure 7A). This depolarization reflected a disruption in mitochondrial membrane potential, a critical early event in the apoptotic process. Mitochondrial dysfunction is a hallmark of apoptosis and an initiator of the intrinsic apoptotic pathway. The opening of the mitochondrial permeability transition pore results in transmembrane potential depolarization, release of apoptogenic factors, and loss of oxidative phosphorylation, ultimately leading to cell death [49,50]. The observed decrease in intracellular calcium levels might be linked to mitochondrial dysfunction, as indicated by the progressive increase in MMP (Figure 7B). Mitochondria regulate intracellular calcium homeostasis by sequestering calcium through the mitochondrial calcium uniporter (MCU). However, excessive MMP elevation can impair calcium uptake by increasing the electrochemical gradient beyond the optimal range for MCU activity, leading to reduced mitochondrial calcium sequestration and lower cytosolic calcium levels [51,52]. This calcium depletion disrupts mitochondrial bioenergetics and compromises ATP production, further exacerbating oxidative stress and apoptosis. Given the essential role of calcium in mitochondrial bioenergetics and cell survival, the interplay between FPN-induced MMP alterations and calcium dysregulation may represent a key event in the immunotoxic effects observed in thymocytes.

Our results demonstrated a significant increase in DCFDA-detected ROS levels at an earlier time point, indicating the rapid induction of oxidative stress following treatment (Figure 8A). This suggests that ROS accumulation serves as an initial trigger for intracellular stress responses, potentially leading to mitochondrial dysfunction, membrane potential collapse, and activation of cell death pathways. The early surge in ROS highlights its pivotal role as a mediator in the cytotoxic effects observed, providing crucial insight into the oxidative stress-driven mechanisms underlying the experimental treatment. Further investigation into antioxidant defense systems and mitochondrial function is warranted to elucidate the downstream effects of this early oxidative response. The broad range of non-target toxicity of FPN is primarily attributed to oxidative stress, which arises when there is an insufficient antioxidant capacity or an accumulation of free radicals [22]. Numerous in vivo and in vitro studies have reported that the generation of ROS or RNS exerts a significant impact on oxidative stress and related toxicities induced by FPN [6,53,54,55,56]. Therefore, the scavenging ability of antioxidants is an effective indicator for assessing oxidative damage from oxidative stimuli. Glutathione (GSH) is the predominant intracellular antioxidant that neutralizes hydroxyl radicals (•OH) and protects against oxidative damage [57]. Variations in GSH levels can significantly affect cellular responses to oxidative stress [58,59].

Our results demonstrate that FPN exposure disrupts intracellular oxidative balance, as reflected by a decline in GSH levels and an increase in lipid peroxidation. The CMFDA assay revealed a significant reduction in GSH levels in the high-dose FPN groups, beginning at the 2-h time point (Figure 7C). This decline corresponds to an early ROS surge, indicating that oxidative stress overwhelmed the cellular redox balance, resulting in the depletion of glutathione reserves. Similarly, the C11-Bodipy^581/591^ assay indicated a pronounced increase in lipid peroxidation at 0.5 and 2 h post-exposure in the high-dose FPN groups (Figure 8B). Lipid peroxidation represents the oxidative degradation of polyunsaturated fatty acids in cellular membranes, causing membrane instability, loss of integrity, and subsequent cellular damage [60,61,62]. These findings suggest that ROS production was elevated by FPN and further affected cellular lipids, thus amplifying oxidative stress.

In the in vitro experiments, we observed time-dependent changes in apoptosis, MMP, and GSH levels in the VH-treated primary thymocytes. Specifically, apoptosis and MMP increased over time, and GSH levels progressively declined. These baseline fluctuations are consistent with the inherent sensitivity of primary thymocytes to in vitro culture conditions. Once isolated from the thymus, thymocytes rapidly undergo apoptosis due to the loss of essential survival signals from thymic stromal cells, cytokines, and other regulatory factors. Additionally, isolation itself induces oxidative stress, as cells are subjected to mechanical and enzymatic processing, shifts in oxygen tension, and changes in nutrient composition. The thymic microenvironment plays a crucial role in maintaining redox homeostasis, and its disruption leads to increased ROS production and antioxidant depletion, as previously reported in studies on oxidative stress following tissue dissociation. Thymic stromal cells, which provide key redox regulatory factors, are absent in vitro, potentially causing elevated ROS levels and compensatory mitochondrial hyperactivity, reflected in MMP changes. Furthermore, the oxygen tension in standard culture conditions is higher than that within the thymus, which may further exacerbate oxidative stress. However, we considered that baseline oxidative stress in VH-treated cells could potentially sensitize them to additional stressors. To address this, we have clarified that, while in vitro conditions contribute to some degree of apoptosis and redox imbalance, the differential effects observed in FPN-treated cells compared with VH-treated cells demonstrated a direct toxic effect of FPN on thymocytes.

Collectively, these findings firmly establish oxidative stress as a critical mechanism mediating FPN-induced immunotoxicity. The observed depletion of GSH directly impairs cellular capacity to neutralize ROS, while the concomitant increase in lipid peroxidation signifies oxidative damage to cellular membranes. These disruptions likely act synergistically, compromising thymocyte survival and disrupting immune homeostasis. This provides a compelling mechanistic explanation for the observed reductions in thymic gene expression and dysregulation of apoptosis. Critically, co-treatment with the antioxidant N-acetylcysteine (NAC) significantly attenuated the deleterious effects of FPN on thymocyte viability and mitochondrial function. As a well-established precursor of GSH, NAC effectively replenishes intracellular GSH levels, thereby mitigating oxidative stress and its downstream consequences.

In this study, NAC co-administration markedly reduced both the FPN-induced surge in ROS and the elevated levels of lipid peroxidation. Furthermore, NAC preserved mitochondrial membrane potential, as evidenced by JC-1 staining, and significantly decreased the proportion of apoptotic cells, as determined by Annexin V/PI staining. These findings strongly support the conclusion that NAC effectively prevents FPN-induced mitochondrial dysfunction and activation of apoptotic pathways. The protective effects of NAC underscore the central role of oxidative stress in FPN-mediated immunotoxicity and highlight the therapeutic potential of antioxidants in mitigating pesticide-induced immune dysfunction.

## 5. Conclusions

The present study demonstrated that fipronil (FPN) exposure disrupted thymic homeostasis by dysregulating key apoptotic and oxidative stress pathways. In vivo, FPN significantly reduced the expression of both anti-apoptotic (*Bcl-2*, *Bcl-6*, *Mcl-1*) and pro-apoptotic (*Bnip3*, *Bim*) genes in thymic tissue, indicating broad transcriptional disruption of apoptotic signaling. In vitro experiments further revealed the direct immunotoxic effects of FPN on mitochondrial dysfunction, characterized by reduced metabolic activity, decreased mitochondrial membrane potential (Δψm), depleted calcium levels, elevated ROS levels, depleted GSH levels, and increased lipid peroxidation (LPO). Notably, in vitro *Bim* expression was significantly increased, suggesting a direct pro-apoptotic response by FPN exposure. Taken together, these findings highlight the critical interplay between oxidative damage and apoptosis in FPN-induced thymocyte toxicity, underscoring the immunotoxic potential of FPN on thymocyte survival and thymus function.

## Figures and Tables

**Table 1:** List of quantitative PCR primers.

Gene Name	Primers (5′ to 3′)
*Bcl-2*	F: CCTGTGGATGACTGAGTACCTG R: AGCCAGGAGAAATCAAACAGAGG
*Mcl-1*	F: AGCTTCATCGAACCATTAGCAGAA R: CCTTCTAGGTCCTGTACGTGGA
*Bcl-6*	F: CAGAGATGTGCCTCCATACTGC R: CTCCTCAGAGAAACGGCAGTCA
*Bnip3*	F: GCTCCAAGAGTTCTCACTGTGAC R: GTTTTTCTCGCCAAAGCTGTGGC
*Bim*	F: GGAGATACGGATTGCACAGGAG R: CTCCATACCAGACGGAAGATAAAG
*Hprt*	F: TCAGTCAACGGGGGACATAAA R: GGGGCTGTACTGCTTAACCAG

## Data Availability

The original data employed or analyzed in this present study can be obtained from the corresponding author upon making a reasonable request.

## Abbreviations

FPN	Fipronil
ConA	Concanavalin A
PMA/Iono	Phorbol 12-myristate 13-acetate/Ionomycin
NAC	N-acetylcysteine
ROS	Reactive oxygen species
RNS	Reactive nitrogen species
MMP	Mitochondrial membrane potential
GSH	Glutathione
LPO	Lipid peroxidation
